# Towards precision medicine in Tourette syndrome: a perspective on AI-driven predictive modelling and personalised care

**DOI:** 10.3389/fncom.2026.1772244

**Published:** 2026-02-19

**Authors:** Cuijie Zhao, Ruixing Li, Lei Hua, Huawei Li, Meng Zhang, Bocai Wang

**Affiliations:** 1 Department of Pediatrics, The First Affiliated Hospital, Henan University of Chinese Medicine, Zhengzhou, Henan, China; 2 School of Pediatrics, Henan University of Chinese Medicine, Zhengzhou, Henan, China

**Keywords:** artificial intelligence, machine learning, personalised treatment, precision medicine, predictive models, Tourette syndrome

## Abstract

Tourette Syndrome (TS) is a complex neurodevelopmental disorder characterised by motor and vocal tics that significantly impair quality of life. Conventional diagnostic and therapeutic methods face challenges due to subjectivity, lack of personalisation, and difficulties in prognostic prediction. Artificial Intelligence (AI) offers novel solutions, advancing TS management towards precision medicine. This article presents a conceptual framework for AI-driven technologies in TS, advocating for a paradigm shift from empirical treatment to precision medicine. We discuss key components including predictive model construction, personalised diagnosis, treatment strategies, and intelligent monitoring. Research indicates that the core value of AI in TS precision medicine lies in its predictiveness, individualisation, and intelligence. Predictive models using multimodal data enable early identification and prognostic assessment. Furthermore, personalised approaches tailor diagnosis and treatment to individual patient characteristics, thereby improving outcomes. Intelligent systems enable automated monitoring and real-time adjustments, optimising clinical workflows. Substantial clinical evidence demonstrates that AI-driven precision medicine improves diagnostic accuracy, optimises treatments, and enhances patient prognosis. Despite this potential, challenges remain in data quality, algorithm interpretability, and clinical translation. Future efforts should focus on enhancing interdisciplinary collaboration, promoting standardisation, and facilitating clinical adoption to deliver more precise, effective, and accessible care for TS patients.

## 1 Introduction

Tourette Syndrome (TS) is a neurodevelopmental disorder characterised by motor and vocal tics. The estimated prevalence of TS is approximately 1/160 (Xiang et al., 2025). TS significantly impacts patients’ quality of life. Furthermore, it frequently co-occurs with conditions such as Attention Deficit Hyperactivity Disorder (ADHD), Obsessive-Compulsive Disorder (OCD), and anxiety. This imposes a considerable burden on patients’ families and society (Martino et al., 2017). The rise of precision medicine and Artificial Intelligence (AI) is transforming TS diagnosis and treatment. The field is shifting from empirical treatments to individualised, intelligent care.

TS diagnosis relies largely on clinical observation and behavioural assessment. However, this subjective approach has limitations. Firstly, symptoms of TS overlap with other neuropsychiatric disorders, including tic disorders, OCD and ADHD, resulting in a high rate of misdiagnosis (Cavanna et al., 2025). Secondly, TS exhibits significant individual variability and heterogeneity, with notable differences in symptom presentation, severity, disease progression, and treatment response among patients (Martino et al., 2017). Thirdly, traditional methods lack objective biomarkers and rely heavily on physician experience. This results in a need for improvement in terms of diagnostic consistency and accuracy. In terms of treatment, therapeutic options for TS are relatively limited, mainly including pharmacological treatment, behavioural therapy and neuromodulation. While pharmacological treatments are effective, they often cause significant side effects and have uncertain long-term outcomes due to inter-individual variability (Frey and Malaty, 2025). Behavioural therapies, including Habit Reversal Training (HRT) and Comprehensive Behavioural Intervention for Tics (CBIT), have been demonstrated to be efficacious. However, the requirement for therapists to be professionally trained limits their accessibility (Frey and Malaty, 2025). Moreover, the lack of effective prognostic tools makes it difficult to predict long-term outcomes and treatment responses, thus impeding the formulation of individualised treatment strategies.

Precision Medicine is a healthcare model that tailors diagnosis and treatment to an individual’s genetic, environmental, and lifestyle differences, with the objective of providing the most suitable solutions for each patient (Kukulka et al., 2025). In the domain of neurodevelopmental disorders, precision medicine employs multi-omics data (genomics, transcriptomics, proteomics, metabolomics, etc.) to achieve a comprehensive understanding of the molecular mechanisms underlying diseases, identify distinct subtypes, predict treatment responses, and facilitate the delivery of personalised therapy (Fukushima-Nomura et al., 2025). AI plays an increasingly vital role in precision medicine, supporting diagnosis, treatment, and prognosis assessment (Plata-Menchaca et al., 2025). AI can process large-scale, multidimensional data to uncover complex patterns that traditional methods miss. In addition, AI technology has been demonstrated to improve the accuracy and efficiency of diagnosis and treatment. Looking forward, AI-driven precision medicine applications in TS should primarily encompass: multimodal diagnostic models based on machine learning (Suganya Devi et al., 2025), individualised drug selection and dosage adjustment (Guo et al., 2025), dynamic treatment plan optimisation based on reinforcement learning (Kachhadia et al., 2025), and prognostic prediction models integrating multi-source data (Liu Y. et al., 2025). These technologies offer novel insights and methodologies for the precise diagnosis and treatment of TS, thereby significantly enhancing diagnostic accuracy and therapeutic outcomes for patients.

The present article aims to outline a roadmap for implementing AI-driven precision medicine in TS, encompassing the latest advancements in predictive model construction, personalised diagnosis, precise treatment strategies, and intelligent monitoring systems. This perspective seeks to synthesise current trends to provide theoretical guidance and technical support for the precise diagnosis and treatment of TS. This perspective provides a comprehensive overview of TS precision medicine, analyses its clinical value and technical challenges, and advocates for interdisciplinary collaboration. We first analyse the need for precision medicine in TS. We then introduce AI-driven technologies for personalised diagnosis and treatment, evaluate their clinical efficacy, and discuss technical challenges. Finally, we summarise future development prospects. The continuous advancement of AI technology and the increasing acceptance of precision medicine indicate that AI-driven TS precision medicine is set to become a pivotal development direction for future TS diagnosis and treatment.

## 2 Analysis of precision medicine needs in Tourette syndrome

As a complex neurodevelopmental disorder, TS exhibits significant individual differences in clinical manifestations, pathological mechanisms, and treatment responses. This provides an important theoretical basis and practical demand for the application of precision medicine. A thorough examination of the characteristics of TS, in conjunction with an analysis of the diagnostic and therapeutic challenges, serves to underscore the imperative and immediacy of the implementation of precision medicine in the context of TS.

### 2.1 Disease heterogeneity and individual differences

The heterogeneity of the disease is reflected at multiple levels, including clinical manifestations, pathological mechanisms, genetic background, and treatment responses. Clinically, TS patients exhibit significant variations in tic symptom types, severity, disease progression, and comorbidities (Groth et al., 2019). Some patients primarily exhibit simple motor tics, while others present with complex motor and vocal tics. Some patients’ symptoms gradually alleviate after adolescence, while others’ symptoms persist into adulthood (Kano et al., 1998).

Pathologically, TS involves abnormalities in multiple neurotransmitter systems, including dopamine, serotonin, and *γ*-aminobutyric acid (GABA; Singer and Pellicciotti, 2025). The efficacy of a given drug treatment can vary significantly between patients, due to the presence of different patterns of neurotransmitter abnormalities. Recent neuroimaging studies have revealed abnormalities in brain structure and function in TS patients, including abnormal regions in the basal ganglia, prefrontal cortex, cingulate gyrus, etc. (Walter et al., 2024).

Genetically, TS shows significant familial aggregation, with a heritability of approximately 0.77 (Yu et al., 2019). Genome-Wide Association Study (GWAS) has identified multiple gene loci associated with TS, including SLITRK1, CNTNAP2, IMMP2L, etc. (Yu et al., 2019). However, these gene loci only explain a small part of the genetic risk of TS, suggesting that the genetic mechanism of TS is extremely complex, involving the interaction of multiple genes and environmental factors.

Regarding treatment, patients demonstrate marked variations in responsiveness to pharmacological interventions. The efficacy of antidepressant medications is contingent on patient response, with some demonstrating responsiveness to dopamine receptor antagonists, while others exhibit a more pronounced response to selective serotonin reuptake inhibitors (SSRIs; Amitai et al., 2013). This individual difference is reflected not only in the selection of drugs, but also in the dosage requirements and sensitivity to side effects.

### 2.2 Limitations of traditional diagnostic and therapeutic methods

Conventional diagnostic and therapeutic methodologies for TS primarily depend on clinical observation of symptoms, behavioural assessment, and empirical treatment, each of which possesses numerous limitations. With regard to the process of diagnosis, the diagnosis of TS is primarily based on the diagnostic criteria set out in the Diagnostic and Statistical Manual of Mental Disorders, 5th Edition (DSM-5) or the International Classification of Diseases, 11th Edition (ICD-11). The aforementioned criteria are predominantly reliant on the description of clinical symptoms and are devoid of objective biomarker support (Pandey et al., 2018). Clinicians must depend on patients’ symptom descriptions and family observations to make a diagnosis, and this subjective diagnostic method is vulnerable to misdiagnosis and missed diagnoses.

With regard to the classification of disease subtypes, conventional TS subtyping is predominantly informed by the severity and nature of symptoms, with a paucity of molecular subtyping based on pathological mechanisms (Pandey et al., 2018). Despite the simplicity and ease of implementation of this phenotypic subtyping method, it is challenging to capture the essential characteristics of the disease, which hinders the development of personalised treatment plans. In terms of treatment, traditional TS treatment mainly adopts a “trial and error” method, that is, first try one treatment method, and if the effect is not good, try other methods (Pandey et al., 2018). This approach is not only inefficient but also has the potential to result in patients missing the optimal treatment opportunity. Furthermore, conventional therapeutic modalities exhibit a paucity of individualised consideration and fail to adequately address patients’ unique characteristics, comorbidities, and treatment preferences. In terms of prognosis assessment, traditional TS prognosis assessment is primarily reliant on the clinical experience of physicians, with an absence of objective prediction tools (Pandey et al., 2018). This complicates the ability of medical professionals to accurately predict patients’ long-term prognoses and to formulate targeted intervention strategies.

### 2.3 Necessity of precision medicine

Given TS’s disease heterogeneity and the limitations of traditional methods, implementing precision medicine is imperative. Precision medicine has the capacity to facilitate a profound comprehension of the molecular mechanisms underlying TS, facilitate the identification of disease subtypes, predict treatment responses, and achieve individualised treatment by integrating multi-omics data (Fukushima-Nomura et al., 2025).

Firstly, the utilisation of precision medicine has the potential to enhance the diagnostic accuracy of TS. The integration of multi-omics information, encompassing genetic data, protein data, and metabolic data, has been demonstrated to facilitate the establishment of more objective and accurate diagnostic models. This, in turn, has the potential to reduce misdiagnosis and missed diagnoses (Li et al., 2025). To illustrate this point, consider the potential of diagnostic models based on machine learning algorithms. These models have the capacity to analyse patients’ clinical features and biomarkers, thereby providing quantitative diagnostic probabilities. Consequently, they can assist clinicians in making more accurate diagnoses.

Secondly, the implementation of precision medicine has the potential to achieve molecular subtyping of TS. By analysing patients’ genotypes, phenotypes, and environmental factors, different disease subtypes can be identified, providing a basis for individualised treatment (Fukushima-Nomura et al., 2025). This molecular subtyping method has the potential to enhance our understanding of the fundamental characteristics of the disease, while also facilitating predictions regarding patients’ treatment responses and prognoses.

Thirdly, the implementation of precision medicine has the potential to enhance the efficacy of TS treatment strategies. By analysing patients’ individual characteristics and treatment history, the effects of different treatment plans can be predicted, providing support for clinical decision-making (Rosenthal et al., 2025). For instance, pharmacogenomic testing can facilitate the selection of drugs and dosages that are optimally suited to an individual patient’s genetic profile, thereby enhancing therapeutic outcomes and mitigating adverse effects.

Fourthly, the implementation of precision medicine has the potential to enhance the evaluation of TS prognosis. Adapting deep learning models used in oncology (Liu Y. et al., 2025), multi-source data integration facilitates accurate long-term outcome prediction. This prediction model has the capacity to provide quantitative risk assessments and to identify the key factors affecting prognosis, thus providing guidance for precise intervention.

### 2.4 Application potential of AI technology

The potential for artificial intelligence (AI) technology in the field of TS precision medicine is significant, as it can address challenges that are challenging to resolve using conventional methods. Furthermore, it has the capacity to enhance the accuracy and efficiency of diagnosis and treatment. The potential of AI technology to be applied in various domains is evident in the following aspects.

With regard to data processing, AI technology has the capacity to process and analyse large-scale, multi-dimensional medical data, including genomic data, transcriptomic data, proteomic data, metabolomic data, imaging data, behavioural data, and so forth (Plata-Menchaca et al., 2025). The data exhibit characteristics of high dimensionality, high noise, and high missing rates, which pose significant challenges to effective processing by traditional data analysis methods. The capacity of AI technology to extract useful information from complex data through the utilisation of deep learning, ensemble learning and other algorithms, and to discover disease-related patterns and regularities, is well-documented.

With regard to pattern recognition, AI technology has been demonstrated to be capable of identifying complex patterns and regularities that are difficult to discover by traditional methods (Suganya Devi et al., 2025). For instance, deep learning algorithms have been shown to be capable of identifying subtle structural changes related to TS from neuroimaging data, which may be difficult for the human eye to detect. Machine learning algorithms have been demonstrated to be capable of identifying gene networks associated with TS from gene expression data, which may involve complex interactions between multiple genes.

In the field of predictive modelling, AI technology has been demonstrated to facilitate the development of more accurate predictive models, encompassing disease risk prediction, treatment response prediction, and prognosis prediction (Li et al., 2025). The integration of multi-source data and consideration of complex nonlinear relationships are capabilities of these predictive models, which consequently yield more accurate prediction results. For instance, predictive models based on gradient boosting algorithms have been shown to predict the incidence risk of TS, and methods based on SHAP value explanation can identify key factors affecting prediction (Li et al., 2025).

With regard to the provision of decision support, AI technology has the capacity to furnish clinicians with intelligent decision support, encompassing diagnostic recommendations, treatment recommendations and prognosis assessment (Guo et al., 2025). These decision support systems have the capacity to provide personalised recommendations based on patients’ individual characteristics and the latest research evidence, thus assisting doctors in making more accurate clinical decisions.

With regard to real-time monitoring, AI technology has been demonstrated to achieve real-time monitoring and automatic adjustment for TS patients (Kachhadia et al., 2025). Through the utilisation of wearable devices, mobile applications, and other technological solutions, it is possible to monitor patients’ symptoms in real-time, automatically adjust treatment plans, and achieve dynamic optimisation.

In summary, AI technology has broad application prospects in TS precision medicine, which can improve the diagnosis and treatment effects of TS in many aspects and provide more precise, effective, and accessible medical services for patients. The ongoing development and refinement of AI technology is poised to unlock a new level of sophistication in its application within the domain of TS precision medicine.

## 3 AI-driven predictive model construction

The construction of predictive models driven by artificial intelligence constitutes a fundamental technology within the domain of precision medicine as practised by TS. The integration of multi-source data facilitates the establishment of accurate predictive models, thereby providing a scientific basis for the diagnosis, treatment and prognosis assessment of TS. In recent years, significant progress has been made in the construction of TS predictive models, driven by the continuous development of machine learning algorithms and the increasing richness of medical data.

### 3.1 Disease risk prediction models

Disease risk prediction models are pivotal in TS precision medicine, identifying high-risk groups to facilitate early intervention. It is acknowledged that risk prediction models based on different data types possess distinct characteristics, and that multimodal data fusion prediction models have the capacity to yield more accurate prediction results.

#### 3.1.1 Gene-based risk prediction

Gene-based risk prediction models principally utilise the results of Genome-Wide Association Studies (GWAS) combined with Polygenic Risk Scores (PRS) to predict the risk of TS onset. In recent years, large-scale GWAS studies have identified multiple gene loci associated with TS, providing an important foundation for risk prediction (Fang et al., 2024). Recent research findings indicate that predictive models founded upon gradient boosting machine learning algorithms have the capacity to effectively predict the risk of TS onset (Muddaloor et al., 2025). In a 2025 study, Li et al. established a TS risk prediction model based on a gradient boosting algorithm by analysing 10 key features, and this model demonstrated good performance in predicting TS onset risk (Li et al., 2025). The study also found that β2-microglobulin and serum 25-hydroxyvitamin D play a critical role in TS risk prediction (Li et al., 2025), providing important biomarkers for early identification of TS (Qiao et al., 2025). Machine learning models have been demonstrated to possess considerable potential in the early identification of neurodevelopmental disorders, with the capacity to enhance diagnostic accuracy and efficiency through multimodal data analysis (Dick et al., 2025).

Gene-based risk prediction models offer several advantages. Firstly, they are able to identify genetic susceptibility, thus providing a basis for the early screening of high-risk populations. Secondly, they can predict the genetic risk of disease, thereby supporting family genetic counselling. Thirdly, they can identify disease-related gene networks, thus providing clues for the discovery of drug targets. However, gene-based predictive models are not without their limitations, including limited heritability, the influence of environmental factors, and complex gene–environment interactions.

#### 3.1.2 Clinical feature-based risk prediction

The primary function of clinical feature-based risk prediction models is the utilisation of information pertaining to patients’ clinical manifestations, family history, and comorbidity status for the purpose of predicting the risk of TS onset. The clinical features in question include tic symptom types, severity, age of onset, disease progression, comorbidity status, and so forth (Dick et al., 2025). In the field of clinical feature prediction, the employment of machine learning algorithms has become a prevalent practice. Such algorithms include, but are not limited to, logistic regression, random forest, and support vector machines. These models have the capacity to analyse complex relationships between clinical features and identify key factors influencing TS onset. For instance, the early onset of complex tic symptoms, a positive family history, and the presence of comorbidity with ADHD or OCD have been shown to be associated with an increased risk of TS onset (Hirschtritt et al., 2015).

The utilisation of clinical feature-based risk prediction models confers several advantages. Primarily, the acquisition of data is relatively uncomplicated, and the clinical operability is robust. Secondly, these models have the capacity to integrate a plethora of clinical information, thereby providing a comprehensive risk assessment. Thirdly, they are capable of providing direct guidance for clinical decision-making. However, it should be noted that these models are not without their limitations. These include strong subjectivity, an absence of objective biomarker support, and limited prediction accuracy.

#### 3.1.3 Environmental factor-based risk prediction

It is evident that environmental factors exert a significant influence on the manifestation of TS, encompassing perinatal factors, infectious factors, and psychosocial factors. The utilisation of environmental factor-based risk prediction models facilitates the identification of environmental risk factors and the provision of guidance for the prevention of TS (Rizwan et al., 2022). It is evident that perinatal factors, including but not limited to premature birth, low birth weight, and intrapartum complications, have been demonstrated to be associated with an increased risk of the onset of TS. It is hypothesised that infectious factors, including but not limited to streptococcal infection and viral infection, may also be associated with the onset of TS. Psychosocial factors, encompassing the family environment, the school environment, and the level of social support, have been demonstrated to exert a significant influence on the onset and progression of TS (Schrag et al., 2019).

The utilisation of multifactorial analysis methods is a customary practice in the development of environmental factor-based risk prediction models. The purpose of this analysis is to quantify the contribution of disparate environmental factors to the onset of TS. These models have the capacity to predict disease risk and provide guidance for environmental interventions.

#### 3.1.4 Multimodal data fusion prediction

Multimodal fusion models integrate genetic, clinical, and environmental data to facilitate comprehensive risk predictions. Similar to approaches in oncology, these models use deep learning to process complex datasets (Mehri-Kakavand et al., 2025). The key technologies for multimodal data fusion include feature selection, data preprocessing, and model fusion. The process of feature selection has been shown to facilitate the identification of the most predictive features, while concomitantly reducing data dimensionality and enhancing model performance. The process of data preprocessing has been shown to be effective in addressing issues such as missing data, outliers, and imbalanced data. Model fusion is a process that combines the prediction results of multiple sub-models with a view to improving overall prediction performance (Wen et al., 2025).

Recent research findings indicate that multimodal data fusion prediction models demonstrate remarkable efficacy in the prediction of TS risk. It is evident that these models have the capacity to provide accurate prediction results. Furthermore, they are able to identify the key factors that influence predictions, thus offering guidance for precise interventions.

### 3.2 Tic symptom severity prediction

Tic symptom severity prediction represents a significant application of TS precision medicine. This capacity encompasses the assessment of patients’ symptom severity, the prediction of changes in symptoms, and the provision of support for treatment decisions.

#### 3.2.1 Tic symptom prediction models

Tic symptom prediction models principally utilise data such as patients’ tic symptom characteristics, disease course information, and treatment history to predict the severity and trends of symptoms. These models typically employ time series analysis, deep learning, and other algorithms (Li et al., 2025). In order to predict symptoms of tic disorder, it is necessary to take into consideration the volatility, periodicity, and environmental sensitivity of said symptoms. For instance, tic symptoms tend to intensify in response to stress, fatigue, and excitement, while they experience a reduction in intensity during periods of relaxation and concentration. In consideration of the aforementioned characteristics, prediction models have been developed that demonstrate the capacity to forecast both short-term and long-term trends in symptoms (Serajee and Mahbubul Huq, 2015). Recent research findings indicate that automatic tic quantification techniques, founded upon computer vision and deep learning methodologies, possess the capacity to accurately identify and quantify motor symptoms, including eye tics (Conelea et al., 2024). These techniques provide new tools for objective assessment and prediction of tic symptoms.

#### 3.2.2 Comorbidity risk prediction

TS patients frequently exhibit a range of comorbid conditions, including, but not limited to, Attention Deficit Hyperactivity Disorder (ADHD), Obsessive Compulsive Disorder (OCD), anxiety, and depression. Comorbidity risk prediction models have been shown to have the capacity to predict patients’ risk of developing comorbidities, thus providing guidance for early intervention (Hirschtritt et al., 2015). The prediction of comorbidity risk is conventionally derived from patients’ clinical characteristics, family history, and environmental factors. For instance, the early onset of ADHD symptoms, a positive family history, and a poor family environment have been found to be associated with an increased risk of comorbidity (Hirschtritt et al., 2015). The implementation of early intervention strategies, predicated on the assessment of comorbidity risk, has been demonstrated to enhance patient prognosis and curtail the prevalence and progression of comorbidities.

#### 3.2.3 Prognostic assessment models

The utilisation of prognostic assessment models has been demonstrated to possess the capacity to predict patients’ long-term outcomes, encompassing the domains of symptom remission, functional recovery, and enhancement in quality of life. These models typically employ survival analysis, machine learning, and other algorithms (Groth et al., 2019). A comprehensive prognostic assessment necessitates the consideration of numerous factors, including the severity of the disease, the response to treatment, the presence of comorbidity, and the social support system available to the patient. The development of prognostic models based on these factors has the potential to provide patients and their families with accurate prognostic information, thereby supporting treatment decisions.

#### 3.2.4 Dynamic prediction systems

Dynamic prediction systems have the capacity to monitor patients’ symptoms in real time, to adjust prediction models in real time, and to provide personalised prediction results. These systems typically employ online learning, reinforcement learning, and other algorithms (Kachhadia et al., 2025). The following technologies are considered to be fundamental to the operation of dynamic prediction systems: real-time data acquisition, model updating, and prediction result output. Through the utilisation of wearable devices, mobile applications, and other technological solutions, it is capable of real-time monitoring of patient symptoms, automated updating of prediction models, and provision of personalised prediction results.

### 3.3 Treatment response prediction

The core application of TS precision medicine is the prediction of treatment response, which is capable of predicting patients’ responses to different treatment regimens and providing a scientific basis for individualised treatment.

#### 3.3.1 Drug response prediction

The primary function of drug response prediction models is to utilise patients’ genotypes, phenotypes, drug metabolism characteristics, and other pertinent information to predict their response to various medications. These models typically employ pharmacogenomics, machine learning, and other methods (Rosenthal et al., 2025). In the field of pharmacology, the prediction of drug response is a significant area of research. A number of factors have been identified as being crucial in this regard, including genes associated with drug metabolism, drug target genes, and genes involved in drug transportation. For instance, CYP2D6 gene polymorphism has been demonstrated to influence the metabolism of various antipsychotic drugs, while COMT gene polymorphism has been shown to impact dopamine metabolism (Rosenthal et al., 2025). The prescription of medication that is tailored to an individual patient’s response to the drug in question has been demonstrated to engender a number of important benefits. These include an improvement in the efficacy of the treatment, a reduction in the incidence of adverse effects, and an enhancement in the quality of life of the patient.

#### 3.3.2 Behavioural therapy response prediction

The utilisation of response prediction models in behavioural therapy facilitates the estimation of patients’ responses to specific therapeutic interventions, thereby providing a framework for the selection of appropriate treatments. These models are typically predicated on patients’ cognitive characteristics, behavioural characteristics, and motivational levels (Serajee and Mahbubul Huq, 2015). A number of factors have been identified as being of key importance in the prediction of responses to behavioural therapy. These include patients’ cognitive abilities, learning abilities, motivational levels, and the level of family support. For instance, patients with superior cognitive abilities tend to demonstrate enhanced responses to cognitive-behavioural therapy, while those with robust family support systems exhibit favourable outcomes following family therapy (Serajee and Mahbubul Huq, 2015).

#### 3.3.3 Neuromodulation therapy response prediction

The development of response prediction models for neuromodulation therapy has enabled the prediction of patient responses to treatments such as deep brain stimulation (DBS) and transcranial magnetic stimulation (TMS). The basis of these models is typically the neuroimaging characteristics, neurophysiological characteristics, and other information of patients (Wehmeyer et al., 2025). The following factors must be considered when predicting the response to neuromodulation therapy: the selection of stimulation targets, the setting of stimulation parameters, and individual patient characteristics. Prediction models based on these factors can provide guidance for the selection and optimisation of neuromodulation therapy.

#### 3.3.4 Integrated treatment strategy prediction

Integrated treatment strategy prediction models have the capacity to predict patients’ responses to different treatment combinations, thereby providing support for the formulation of comprehensive treatment plans. These models typically employ multi-objective optimisation, reinforcement learning, and other algorithms (Kachhadia et al., 2025). The development of an integrated treatment strategy necessitates a comprehensive consideration of the interactions between various treatments, the individual characteristics of patients, and the treatment objectives. Prediction models based on these factors can provide a scientific basis for the formulation and optimisation of integrated treatment plans.

### 3.4 Multimodal data integration strategies

Multimodal data integration is a pivotal technology in the field of TS precision medicine, with the capacity to amalgamate data from disparate sources, thereby yielding more comprehensive and accurate information.

#### 3.4.1 Genomic data integration

Genomic integration combines genotype, expression, and epigenetic data to provide comprehensive genetic insights. These data can provide disease-related genetic information, thus establishing a foundation for precision medicine (Fang et al., 2024). The integration of genomic data is contingent upon the implementation of three fundamental technologies: data standardisation, quality control, and variant annotation. The integration of multiple genomic data sets facilitates the identification of disease-related gene networks, thereby providing a foundation for the identification of drug targets.

#### 3.4.2 Clinical data integration

Clinical data integration merges symptoms, signs, and laboratory results to support robust diagnosis. These data can provide disease-related clinical information, thereby supporting diagnosis and treatment (Li et al., 2025). The following technologies are pivotal for clinical data integration: data cleaning, feature engineering, and data fusion. The integration of multiple clinical data sets has been demonstrated to facilitate the establishment of more accurate diagnostic and treatment models.

#### 3.4.3 Imaging data integration

Imaging integration unifies structural, functional, and diffusion MRI data, offering objective neuroimaging evidence. These data can provide disease-related neuroimaging information, thereby offering an objective basis for diagnosis and treatment (Walter et al., 2024). The following technologies are considered to be of key importance with regard to the integration of imaging data: image preprocessing, feature extraction and multimodal fusion. The integration of multiple imaging data sources facilitates the identification of disease-related neuroimaging biomarkers, thereby supporting precise diagnostic decisions.

#### 3.4.4 Behavioural data integration

Behavioural integration incorporates observational data, questionnaires, and cognitive tests. These data can provide disease-related behavioural information, thereby serving as a reference for diagnosis and treatment (Serajee and Mahbubul Huq, 2015). The following technologies are considered to be fundamental to the integration of behavioural data: data standardisation, feature extraction and behavioural modelling. The integration of multiple behavioural data sources has been demonstrated to facilitate the development of more accurate behavioural assessment models.

#### 3.4.5 Environmental data integration

Environmental integration aggregates exposure, lifestyle, and social factors to identify external risk contributors. These data can provide disease-related environmental information, thereby providing guidance for prevention and intervention (Schrag et al., 2019). The following technologies are considered to be of key importance with regard to the integration of environmental data: firstly, data collection, secondly, data cleaning, and thirdly, environmental modelling. The integration of multiple environmental data sets facilitates the identification of environmental risk factors, thereby providing a scientific foundation for environmental interventions. In summary, multimodal data integration strategies provide substantial technical support for precision medicine in TS, capable of integrating data from different sources, providing more comprehensive and accurate information, and offering a scientific basis for precise diagnosis, treatment, and prognosis assessment.

## 4 AI-driven personalised diagnosis and precision treatment

The application of TS precision medicine is centred on two core concepts: AI-driven personalised diagnosis and precision treatment. The integration of multi-source data with intelligent diagnostic and therapeutic systems facilitates the establishment of individualised medical services for patients suffering from TS. This technological framework has been demonstrated to enhance the accuracy and efficiency of diagnosis, while also facilitating individualised precision treatment, thereby leading to a substantial improvement in patient prognosis.

### 4.1 Intelligent diagnostic systems

Intelligent diagnostic systems represent a pivotal component of AI-driven TS precision medicine, demonstrating the capability to deliver precise, objective, and expeditious diagnostic outcomes through the analysis of multimodal data. These systems typically employ machine learning, deep learning, and other algorithms to identify diagnostic features that are difficult for traditional methods to discover.

#### 4.1.1 Symptom-based intelligent diagnosis

Symptom-based intelligent diagnostic systems principally employ data comprising patients’ clinical symptoms, behavioural manifestations and disease course information for the purpose of establishing diagnostic models. These systems typically employ natural language processing, machine learning, and other algorithms to extract key information from symptom descriptions and establish diagnostic rules (Schappert et al., 2024). Recent research demonstrates the capacity of machine learning-based symptom analysis systems to accurately identify typical TS symptom patterns, including characteristics, frequency, and severity of motor and vocal tics. These systems have the capacity to provide quantitative symptom assessment and to identify relationships between symptoms, thus offering a more objective basis for diagnosis (Chu et al., 2023). The utilisation of symptom-based intelligent diagnostic systems confers several advantages. Primarily, these systems have the capacity to standardise the assessment of symptoms, thereby reducing subjectivity. Secondly, they are able to identify complex symptom patterns, thus enhancing diagnostic accuracy. Thirdly, these systems can provide quantitative symptom scores, which facilitates disease monitoring and treatment efficacy assessment.

#### 4.1.2 Imaging-based intelligent diagnosis

Imaging-based systems use neuroimaging data to identify structural and functional abnormalities. These systems typically employ deep learning, convolutional neural networks, and other algorithms to extract features from imaging data and establish diagnostic models (Huang et al., 2025). Neuroimaging studies have demonstrated that patients diagnosed with Tourette syndrome (TS) exhibit abnormalities in multiple brain regions, including the basal ganglia, prefrontal cortex, cingulate gyrus, and cerebellum. Deep learning-based imaging analysis systems have been shown to accurately identify these abnormalities, providing objective imaging evidence for TS diagnosis (Liu et al., 2013). Recent research demonstrates the efficacy of automated video analysis techniques based on computer vision and deep learning in accurately identifying and quantifying movement disorders, thus providing novel tools for TS diagnosis (Pecoraro et al., 2025). These techniques have been shown to not only identify obvious tic symptoms but also subtle movement abnormalities, thus improving diagnostic sensitivity.

#### 4.1.3 Biomarker-based diagnosis

Biomarker-based diagnostic systems primarily utilise molecular biomarkers in biological samples, such as blood, cerebrospinal fluid, and urine, to establish diagnostic models. These systems generally utilise proteomics, metabolomics, genomics, and other technologies to identify disease-related biomarkers (Lei et al., 2012). Recent research findings indicate that biomarkers such as β2-microglobulin and serum 25-hydroxyvitamin D possess significant diagnostic value in the context of TS (Li et al., 2025). The utilisation of diagnostic models founded upon these biomarkers has the potential to furnish objective diagnostic evidence, thereby diminishing reliance on clinical symptoms. The utilisation of biomarker-based diagnostic systems confers several advantages. Primarily, these systems offer objective diagnostic evidence, thereby reducing subjectivity. Secondly, they facilitate early diagnosis, thus enhancing diagnostic timeliness. Thirdly, they enable the monitoring of disease progression and the evaluation of treatment efficacy.

#### 4.1.4 Multimodal diagnostic fusion

Multimodal diagnostic fusion systems have the capacity to integrate a variety of data types, including symptom data, imaging data and biomarker data, thereby producing more accurate and comprehensive diagnostic results (Gao et al., 2025). These systems typically employ multimodal learning, ensemble learning, and other algorithms to fully utilise the complementarity of different data modalities (Mehri-Kakavand et al., 2025). The technologies that are pivotal to multimodal diagnostic fusion can be categorised into three distinct areas: firstly, feature extraction, secondly, feature fusion, and finally, model ensemble. The process of feature extraction is capable of extracting useful features from different modal data (Samadi Miandoab et al., 2025). Feature fusion is able to integrate features from different modalities to form a unified feature representation (Zheng et al., 2025). Model ensemble is able to combine the prediction results of multiple sub-models to improve overall diagnostic performance (Wen et al., 2025). Recent research indicates that multimodal fusion-based diagnostic systems demonstrate excellent performance in the domain of TS diagnosis, exhibiting the capacity to provide accurate diagnostic results and to identify the key factors influencing diagnosis, thereby offering guidance for precise diagnosis (Wen et al., 2018).

### 4.2 Precision subtyping and early identification

Precision subtyping and early identification represent significant applications of TS precision medicine, with the capacity to identify diverse disease subtypes and facilitate early screening and intervention for high-risk demographics.

#### 4.2.1 Phenotype-based subtyping

Phenotype-based subtyping systems principally utilise information such as patients’ clinical manifestations, behavioural characteristics, and comorbidity status to identify different disease subtypes. These systems generally utilise cluster analysis, machine learning, and other algorithms to identify patient groups exhibiting analogous phenotypic characteristics (Hirschtritt et al., 2015). Typically, TS phenotypic subtyping is based on factors such as tic symptom type, severity, age of onset, disease progression, and comorbidity status. For instance, some patients primarily exhibit motor tics, some primarily vocal tics, and others exhibit a combination of both (Kano et al., 1998). Phenotype-based subtyping systems have the potential to provide a basis for individualised treatment, as patients with different subtypes may require different treatment strategies.

#### 4.2.2 Genotype-based subtyping

Genotype-based subtyping uses genetic information to identify disease subtypes (Fang et al., 2024). Recent studies, including those using CRISPR/Cas9 mouse models (Nasello et al., 2024), underpin this approach. The present studies provide an important theoretical basis for genotype-based TS subtyping. Genotype-based subtyping systems have the potential to provide a basis for precision treatment, as patients with different genotypes may respond differently to various therapeutic drugs.

#### 4.2.3 High-risk population screening and early symptom identification

The implementation of high-risk population screening and early symptom identification systems has the potential to identify individuals within high-risk populations for TS, thereby facilitating early intervention. These systems generally evaluate the risk of TS onset by considering family history, genotype, environmental factors, and other pertinent information (Yu et al., 2019). The following factors must be considered when conducting high-risk population screening: a positive family history, a high-risk genotype, and adverse environmental factors. Screening systems based on these factors have the capacity to identify high-risk populations and provide guidance for early intervention. Early symptom identification systems have been developed for the purpose of identifying early symptoms of TS, including subtle motor abnormalities, behavioural changes, and cognitive dysfunction. These systems typically employ machine learning, deep learning, and other algorithms to identify early signs of TS from complex symptom patterns (Tang et al., 2024).

#### 4.2.4 Dynamic subtyping and early warning systems

The utilisation of dynamic subtyping methodologies in conjunction with the implementation of early warning systems facilitates the real-time monitoring of patient disease progression, enables the dynamic adjustment of subtyping results, and provides early warning information. These systems typically employ online learning, reinforcement learning, and other algorithms to dynamically update models based on new data (Bernabei et al., 2010). Dynamic subtyping systems have the capacity to adjust patients’ disease subtypes in real time, based on factors such as disease changes, treatment response, and environmental factors. This provides a degree of individualised guidance for treatment. Early warning systems have the capacity to monitor patients’ disease changes and promptly detect abnormalities such as disease deterioration, poor treatment response, and side effects, providing early warnings for clinical intervention (Morishita et al., 2022).

### 4.3 Personalised treatment strategies

Personalised treatment strategies represent a fundamental aspect of TS precision medicine, with the capacity to formulate the most suitable treatment plans based on patients’ individual characteristics. This approach has been shown to enhance treatment efficacy and reduce adverse effects.

#### 4.3.1 Precise drug selection and dosage adjustment

Precise drug selection and dosage adjustment systems have the capacity to select the most suitable drugs and dosages based on patients’ genotypes, phenotypes, drug metabolism characteristics, and other information. These systems generally utilise pharmacogenomics, machine learning, and other methodologies (Mehanna et al., 2013). The selection of pharmaceuticals is contingent upon a number of factors, including patients’ genotypes, the activity of drug-metabolising enzymes, the sensitivity of drug targets, and the presence of comorbidity. For instance, CYP2D6 gene polymorphism has been demonstrated to influence the metabolism of various antipsychotic drugs, while COMT gene polymorphism has been shown to impact dopamine metabolism (Chevreuil et al., 2011). Dosage adjustment must take into account patients’ individual characteristics, drug metabolism capacity, treatment response, and sensitivity to side effects. Systems for adjusting dosages based on these factors have the potential to enhance the efficacy of pharmaceutical treatments while concomitantly reducing the incidence of adverse effects.

#### 4.3.2 Genotype and phenotype-based drug selection

Genotype and phenotype-based drug selection systems have the capacity to select the most suitable drugs for patients based on their genotypes and phenotypic characteristics. These systems generally utilise pharmacogenomics, phenomics, and other technologies to predict patients’ responses to different drugs (Coulter et al., 2025). Genotype information encompasses drug-metabolising enzyme genes, drug target genes, and drug transporter genes. Phenotype information encompasses patients’ clinical manifestations, behavioural characteristics and cognitive function. The utilisation of such information in the development of drug selection systems has the potential to provide a scientific basis for the administration of medication tailored to the individual patient. Recent research demonstrates that pharmacogenomics-based individualised medication can significantly improve the treatment efficacy of TS patients, reduce side effects, and enhance quality of life (Tavakoli et al., 2025).

#### 4.3.3 Drug interaction prediction

The capacity of drug interaction prediction systems to predict interactions between different drugs is well documented, and the guidance they provide for combination medication is widely accepted. These systems generally employ drug interaction databases, machine learning, and other methods to identify potential drug interactions (Marzouk et al., 2025). The following factors are considered to be of key importance in the process of predicting interactions between drugs: drug metabolism pathways, mechanisms of action, and pharmacokinetic characteristics. Prediction systems based on these factors have the capacity to identify potential drug interactions and ensure the safety of combination medication.

#### 4.3.4 Multi-objective optimisation and dynamic adjustment

Multi-objective optimisation and dynamic adjustment systems have the capacity to simultaneously enhance multiple treatment objectives, including, but not limited to, symptom remission, minimisation of adverse effects, and enhancement in quality of life. These systems generally utilise multi-objective optimisation, reinforcement learning, and other algorithms to achieve dynamic optimisation of treatment plans (Shuvo et al., 2023). Multi-objective optimisation necessitates the consideration of trade-offs between disparate objectives, such as the balance between treatment efficacy and side effects. Dynamic adjustment systems have the capacity to adapt treatment plans in real-time, utilising patients’ treatment responses, disease progression, and other data to ensure personalised treatment.

### 4.4 Intelligent monitoring and optimisation

Intelligent monitoring and optimisation systems have the capacity to monitor patients’ disease changes in real time, automatically adjust treatment plans, and achieve dynamic optimisation.

#### 4.4.1 Personalised cognitive training and behavioural intervention

Personalised cognitive training and behavioural intervention systems have the capacity to formulate individualised training and intervention plans based on patients’ cognitive characteristics, behavioural characteristics, and learning abilities. These systems generally employ cognitive science, behavioural science, machine learning, and other methods (Serajee and Mahbubul Huq, 2015). Cognitive training encompasses a range of components, including attention training, executive function training, and working memory training. Behavioural interventions encompass habit reversal training, comprehensive behavioural intervention, and cognitive behavioural therapy. The efficacy of treatment can be enhanced through the provision of personalised training and intervention programmes, which are tailored to the individual characteristics of each patient.

#### 4.4.2 Virtual reality therapy

Virtual reality therapy systems have the capacity to utilise virtual reality technology in order to provide immersive treatment environments for patients diagnosed with TS. These systems generally utilise virtual reality technology, human-computer interaction technology, machine learning, and other methodologies to facilitate personalised virtual treatment experiences (Single et al., 2025). The advantages of virtual reality (VR) therapy are manifold. In terms of the treatment environment, it is safe and controllable. Secondly, it is able to simulate real-life scenarios, thus improving the practicality of treatment. Thirdly, it can provide personalised treatment content to meet the needs of different patients.

#### 4.4.3 Real-time monitoring and automatic adjustment

Real-time monitoring and automatic adjustment systems have the capacity to monitor patients’ symptoms in real-time and automatically adjust treatment plans through wearable devices, mobile applications, and other technologies. These systems generally employ Internet of Things (IoT) technology, machine learning, reinforcement learning, and other methods (Ganatra et al., 2025). The following technologies are pivotal for the real-time monitoring of systems: sensor technology, data transmission technology and data processing technology. Automatic adjustment systems have the capacity to adjust drug dosages, treatment parameters, intervention strategies, and so forth, automatically and based on monitoring data. This process is known as dynamic optimisation.

#### 4.4.4 Combined treatment strategy optimisation

The application of combined treatment strategy optimisation systems has been demonstrated to enhance the efficacy of diverse treatment methods, thereby achieving synergistic outcomes. These systems generally utilise multi-objective optimisation, systems biology, machine learning, and other methodologies to ascertain the most efficacious treatment combination (Kang et al., 2025). The optimisation of combined treatment strategies necessitates the consideration of interactions between disparate treatment methods, individual patient characteristics, and treatment objectives. Optimisation systems based on these factors can provide a scientific basis for the formulation of comprehensive treatment plans.

### 4.5 AI technology core and algorithm selection

The technical foundation of TS precision medicine is constituted by two key elements: AI technology core and algorithm selection. The selection of an appropriate algorithm is of paramount importance for enhancing system performance, with different algorithms proving suitable for different application scenarios.

#### 4.5.1 Machine learning algorithm selection

The selection of machine learning algorithms necessitates the consideration of several factors, including the data type, the scale of the data, the prediction target, and the computational resources available. Machine learning algorithms that are frequently employed include logistic regression, random forest, support vector machine, and gradient boosting (Kiremit and Şahin, 2025). Logistic regression is considered to be suitable for classification problems with strong linear relationships; random forest is considered to be suitable for high-dimensional data, feature selection, and model interpretability; support vector machine is considered to be suitable for small samples, high-dimensional data, and nonlinear classification; gradient boosting is considered to be suitable for complex nonlinear relationships and high-precision prediction (Meaney et al., 2025).

#### 4.5.2 Deep learning model application

The potential of deep learning models to enhance the precision of TS medicine is significant. These models have the capacity to manage intricate nonlinear relationships, high-dimensional data, and multimodal data, which is a crucial aspect of modern healthcare. The following deep learning models are frequently employed: convolutional neural networks, recurrent neural networks and Transformers (Suganya Devi et al., 2025). Convolutional neural networks are well-suited for image data and sequence data; recurrent neural networks are well-suited for time series data and natural language processing; Transformers are well-suited for sequence data and multimodal data. The selection of appropriate deep learning models necessitates the consideration of data characteristics, task requirements and computational resources.

#### 4.5.3 Multimodal fusion technology

Multimodal fusion technology is a pivotal component of TS precision medicine, with the capacity to amalgamate data from disparate sources, thereby yielding more comprehensive and accurate information. The following multimodal fusion technologies are most commonly utilised: early fusion, late fusion, and hybrid fusion (Mehri-Kakavand et al., 2025). Early fusion performs fusion at the feature level, fully leveraging the complementarity of different modal data; late fusion performs fusion at the decision level, maintaining the independence of different modal data; hybrid fusion combines the advantages of early and late fusion, providing enhanced fusion effects.

#### 4.5.4 Real-time processing systems

Real-time processing systems represent a significant technological advancement, providing a crucial support framework for precision medicine in TS, encompassing real-time data acquisition, processing, analysis, and decision-making capabilities. These systems typically employ a combination of technologies, including streaming processing, edge computing, and cloud computing (Thilagaraj et al., 2022). The following technologies are of particular significance in the domain of real-time processing systems: data stream processing, real-time computing, and low-latency communication. The optimisation of these technologies has the potential to achieve millisecond-level data processing and decision response, thereby providing technical support for real-time monitoring and automatic adjustment. In summary, artificial intelligence (AI)-driven personalised diagnosis and precision treatment provide strong technical support for TS precision medicine, enabling individualised diagnosis, treatment, and monitoring, and significantly improving patient prognosis. The ongoing development and refinement of AI technology is poised to unlock a new level of sophistication in its application within the domain of TS precision medicine.

## 5 Clinical efficacy and validation studies

AI-driven precision medicine technologies for TS have demonstrated considerable advantages in clinical applications, with the capacity to enhance diagnostic accuracy, augment treatment efficacy, and optimise prognostic assessment. This chapter will systematically analyse the results of relevant clinical studies to evaluate the clinical efficacy and value of AI technology in TS precision medicine.

### 5.1 Improvement in diagnostic accuracy

#### 5.1.1 Comparison of diagnostic accuracy rates

A substantial body of research has demonstrated that AI-based TS diagnostic systems exhibit a marked superiority in terms of diagnostic accuracy when compared with conventional diagnostic methodologies. The present study employed a retrospective observational study design to make a comparison between the performance of traditional diagnostic methods and machine learning-based predictive models in the context of TS diagnosis. For example, gradient boosting models have shown improved discriminatory performance compared with traditional approaches in TS-related prediction tasks (Li et al., 2025). Multimodal data fusion diagnostic systems demonstrated a heightened level of diagnostic accuracy. These systems have the capacity to integrate a variety of data types, including symptom data, imaging data and biomarker data, thereby producing more accurate and comprehensive diagnostic results. In other complex disorders such as non-small cell lung cancer, multimodal fusion systems have demonstrated robust performance (Mehri-Kakavand et al., 2025), suggesting potential value for TS applications.

#### 5.1.2 Shortening of diagnosis time

The utilisation of AI technology has the potential to markedly reduce the diagnostic time of TS, thereby enhancing diagnostic efficiency. Conventional TS diagnosis is typically characterised by the necessity of multiple visits and prolonged observation periods, consequently leading to protracted diagnostic times. AI-based diagnostic systems have the capacity to rapidly analyse patients’ symptom characteristics and provide preliminary diagnostic results (Schappert et al., 2024). Machine learning-based symptom analysis systems can support rapid quantitative assessment in appropriate settings. Imaging-based intelligent diagnostic systems have the capacity to complete imaging analysis within hours, thereby providing objective imaging diagnostic evidence. Biomarker-based diagnostic systems may accelerate the identification of candidate biomarkers and provide more objective evidence to complement symptom-based assessment (Conelea et al., 2024). Multimodal fusion diagnostic systems have the capacity to process various types of data concurrently, thereby reducing the time required for diagnosis. Multimodal fusion systems may streamline diagnostic workflows by integrating heterogeneous data sources (Zubair et al., 2025).

#### 5.1.3 Reduction in misdiagnosis rate

AI reduces misdiagnosis rates by identifying characteristic symptom patterns. The prevailing approach to TS diagnosis is predicated on clinical observation of symptoms, a method that is susceptible to misdiagnosis due to its similarity to other neuropsychiatric disorders (Szejko and Muller-Vahl, 2021). The utilisation of artificial intelligence (AI) in the development of diagnostic systems has enabled the identification of characteristic symptom patterns associated with TS, thereby facilitating its differentiation from other diseases. Machine learning-based symptom analysis systems have been shown to accurately identify the characteristics of motor and vocal tics, thereby reducing confusion with other movement disorders. The utilisation of imaging-based intelligent diagnostic systems facilitates the identification of structural and functional abnormalities within the brain that are associated with TS, thereby providing objective diagnostic evidence (Wen et al., 2018). The utilisation of biomarker-based diagnostic systems has the potential to offer objective diagnostic evidence, thereby reducing reliance on clinical symptoms. Research has demonstrated that diagnostic models founded upon biomarkers such as β2-microglobulin and serum 25-hydroxyvitamin D have the capacity to markedly curtail the incidence of misdiagnosis (Jiang et al., 2024).

#### 5.1.4 Improvement in diagnostic consistency

The application of AI technology has the potential to enhance the consistency of TS diagnosis and reduce diagnostic differences among different physicians. The prevailing approach to TS diagnosis is predicated on the expertise of medical practitioners, with the acknowledgement that diagnostic outcomes can vary among different healthcare professionals (Verrel et al., 2026). The utilisation of artificial intelligence (AI) in diagnostic systems has the potential to standardise procedures, thereby reducing the element of subjectivity. Machine learning-based symptom analysis systems have the capacity to provide quantitative symptom scores, thereby standardising the process of symptom assessment. The utilisation of imaging-based intelligent diagnostic systems has been demonstrated to facilitate the provision of objective imaging diagnostic evidence, thereby reducing subjectivity (de Barros et al., 2025). Multimodal fusion diagnostic systems have the capacity to integrate a variety of diagnostic information, thereby producing more comprehensive and consistent diagnostic results. Research has demonstrated that multimodal fusion-based diagnostic systems significantly enhance diagnostic consistency among different physicians (Zhang et al., 2025).

### 5.2 Improvement in treatment efficacy

The utilisation of AI technology in TS treatment has the potential to enhance treatment efficacy, thereby increasing treatment success rates and patient satisfaction.

#### 5.2.1 Improvement in treatment success rate

AI-driven personalised strategies enhance treatment efficacy. Conventional TS treatment is chiefly characterised by a “trial and error” approach, resulting in treatment success rates that are considered to be limited. AI-based treatment systems have the capacity to select the most suitable treatment plans based on patients’ individual characteristics (Higgins et al., 2025). The implementation of pharmacogenomic-based individualised medication has been demonstrated to result in a substantial enhancement in the efficacy of pharmacological treatment. For example, pharmacogenetic testing has been evaluated as a strategy to guide antipsychotic prescribing and improve stratification in psychiatric care (Saadullah Khani et al., 2024), a model that may inform individualised medication selection in TS. Machine learning-based treatment response prediction models have the capacity to predict patients’ responses to a range of treatment regimens, thereby providing a framework for the selection of appropriate treatments. Machine learning models have been developed to predict psychotherapy outcomes using clinical and neuroimaging data, supporting more individualised treatment planning in neuropsychiatric disorders (van de Mortel et al., 2025).

#### 5.2.2 Increase in symptom remission rate

The utilisation of AI technology has the potential to enhance the efficacy of treatment, leading to a higher rate of symptom remission in patients diagnosed with TS. Furthermore, the integration of AI-driven solutions can contribute to an improvement in the quality of life for these individuals. Digital and app-assisted behavioural interventions can support more individualised treatment delivery for tic disorders, with early trials suggesting feasibility and potential clinical benefit (Johnk et al., 2025). The utilisation of a multimodal data fusion-based treatment strategy optimisation approach has the capacity to identify optimal treatment combinations, thereby increasing the rate of symptom remission. Internet-delivered behavioural interventions for paediatric TS have demonstrated sustained clinical benefit in randomised evaluations, supporting scalable, personalised care models (Andren et al., 2024). Intelligent treatment systems that are based on real-time monitoring and automatic adjustment have the capacity to dynamically optimise treatment plans, thereby improving treatment efficacy. Closed-loop neuromodulation frameworks incorporate real-time biomarkers to adjust stimulation parameters, offering a pathway towards adaptive, individualised symptom control in selected neuropsychiatric conditions (Balachandar et al., 2024).

#### 5.2.3 Reduction in side effects

The utilisation of AI technology has the potential to substantially mitigate the adverse effects associated with TS treatment and enhance its safety profile. Conventional TS treatment has been observed to result in a number of adverse effects, which can potentially compromise patient adherence to the prescribed treatment regimen (Panda et al., 2025). Pharmacogenomics-based individualised medication is a process which selects appropriate drugs and dosages based on patients’ genotypes, with the objective of reducing side effects. Pharmacogenomics-guided drug and dosage selection can significantly reduce drug-gene-related adverse reactions (Tsermpini et al., 2020). Machine learning-based side effect prediction models have been developed to predict patients’ risk of developing side effects, thus providing guidance for treatment selection. Research has demonstrated that a treatment plan that is tailored to the individual, with the aim of predicting potential side effects, has the capacity to reduce the incidence of such adverse reactions by more than 30% (Guo and Choo, 2025).

#### 5.2.4 Improvement in patient satisfaction

The implementation of AI technology has the potential to enhance TS patient satisfaction and improve the patient experience. AI-based precision treatment has the capacity to provide personalised treatment plans that meet the needs of different patients (Dutta and Das, 2025). Virtual reality-based treatment systems have the capacity to provide an immersive experience for patients, increasing patient engagement and satisfaction. Virtual reality–based interventions may improve patient engagement and satisfaction in selected rehabilitation contexts (Jo et al., 2024). Intelligent monitoring and automatic adjustment-based treatment systems have the capacity to monitor patients’ disease changes in real time and automatically adjust treatment plans, thus enhancing the patient experience. Remote care programmes incorporating monitoring and follow-up may support patient satisfaction and perceived usefulness (Johnk et al., 2025).

### 5.3 Prognosis improvement effects

The implementation of AI technology in the assessment of TS prognosis has the potential to engender substantial improvements in patient prognosis, alongside the enhancement of patients’ quality of life and social function.

#### 5.3.1 Long-term prognosis improvement

The utilisation of artificial intelligence (AI)-based prognostic assessment models has been demonstrated to facilitate precise prediction of patients’ long-term outcomes, thereby providing a framework for personalised intervention strategies. The conventional TS prognostic evaluation predominantly depends on the clinical expertise of physicians, exhibiting suboptimal precision (Santurro et al., 2025). Multimodal data fusion-based prognostic prediction models have the capacity to integrate a variety of prognostic factors, thereby facilitating more accurate prognostic assessments. Research has demonstrated that multimodal fusion-based prognostic prediction models demonstrate excellent performance in the prediction of long-term prognosis (Chen et al., 2025). Machine learning-based prognostic assessment models have the capacity to identify the key factors affecting prognosis, thereby providing guidance for precise intervention. Research has demonstrated that a patient-centred intervention, tailored to their individual prognosis, has the capacity to markedly enhance long-term prognoses (Groth, 2018).

#### 5.3.2 Improvement in quality of life

The utilisation of AI technology has the potential to markedly enhance the quality of life for TS patients, thereby promoting optimal health and well-being. AI-based precision treatment has been demonstrated to effectively control symptoms and reduce the impact of the disease on patients’ lives (Wang et al., 2025). Treatment systems founded upon personalised cognitive training and behavioural intervention have been demonstrated to enhance patients’ cognitive function and behavioural performance, thereby improving quality of life. Personalised training programmes may improve functional outcomes and aspects of quality of life, although effect sizes can vary across populations and protocols (van de Griendt et al., 2024). Intelligent monitoring and automatic adjustment-based treatment systems have the capacity to monitor patients’ disease changes in real time and to promptly adjust treatment plans, thus improving quality of life. Research indicates that treatment plans based on intelligent monitoring may improve quality of life by enabling earlier adjustments and more consistent support (Hollis et al., 2023).

#### 5.3.3 Improvement in social function

The utilisation of AI technology has the potential to enhance the social function of TS patients and promote increased social participation. TS patients frequently encounter difficulties in social adaptation as a result of the impact of symptoms on their capacity for social function (Jiang et al., 2025). A comprehensive intervention, incorporating a personalised treatment approach, has been demonstrated to enhance patients’ social function and promote social adaptability. Research has demonstrated that comprehensive interventions based on individualised treatment may enhance social functioning and participation by supporting more personalised and sustained interventions (Hartmann et al., 2026). Intelligent monitoring and automatic adjustment-based treatment systems have the capacity to monitor changes in patients’ social function in real time and to promptly adjust intervention strategies, thus improving social function. Research has demonstrated that intelligent monitoring-based treatment regimens may support social participation by reducing symptom burden and enabling adaptive follow-up (Green et al., 2025).

#### 5.3.4 Reduction in economic burden

The utilisation of AI technology has the potential to substantially alleviate the financial burden on TS patients, thereby enhancing the cost-effectiveness of treatment. The financial implications of conventional TS treatment can be substantial, resulting in a considerable economic burden on patients’ families (Wanga et al., 2025). The utilisation of AI-based precision treatment has been demonstrated to enhance treatment efficacy, reduce treatment time, and decrease treatment costs. Precision-guided interventions may reduce unnecessary resource use and improve cost-effectiveness, depending on implementation context (Uthman et al., 2025). Intelligent monitoring and automatic adjustment-based treatment systems have the capacity to reduce the number of unnecessary examinations and treatments, thereby lowering medical costs. Research has demonstrated that intelligent monitoring-based treatment may reduce unnecessary visits and procedures and improve efficiency, with cost impacts varying by setting (Hollis et al., 2023)^91^.

### 5.4 Summary of clinical evidence and critical analysis

We have summarised the key evidence regarding the clinical efficacy and validity of AI-driven technologies for TS in Table 1. A critical review of these studies reveals that the application of AI in TS is currently transitioning from proof-of-concept experimental models to early clinical validation.

**Table 1:** Overview of representative studies.

Author	Year	Sample size	Methodology	Main results	Limitations	Clinical validation status
Li A et al.	2025	*N* = 671	Retrospective observational	Diagnosis/Prediction: ML risk prediction for TS; SHAP supports interpretable feature contribution (risk stratification potential).	Retrospective single-centre; nonspecific biomarkers; confounding/limited specificity	TS-specific, early clinical evidence
Mehri-Kakavand G et al.	2025	*N* = 131	Retrospective observational	Prognosis (cross-domain exemplar): multimodal radiomics fusion improves recurrence prediction workflows; informs TS multimodal fusion framing.	Not TS; generalizability to TS unknown	Cross-domain exemplar
Schappert R et al.	2024	*N* = 42	Retrospective observational	Diagnosis: automated video-based assessment supports objective/efficient tic evaluation (clinical workflow automation).	Limited tic coverage; context-dependent; subtle tics underdetected	TS-specific, under development/early
Conelea C et al.	2024	*N* = 54	Retrospective observational	Monitoring/Diagnosis: computer vision + DL quantify eye tics to reduce subjective rating burden and enable scalable assessment.	Small sample; limited generalizability; no control/other movement-disorder comparators	TS-specific, early clinical evidence
Zubair M et al.	2025	N/A	Review	Diagnosis (methodological): summarises multimodal image fusion techniques that can be adapted for TS imaging pipelines.	Not TS-specific; heterogeneous evidence	Cross-domain methods review
Wen H et al.	2018	TS (*N* = 29); Normal (*N* = 37)	Retrospective observational	Diagnosis: ML on fMRI functional connectivity supports TS classification (evidence for neuroimaging biomarkers).	Small sample; external validation NR	TS-specific, early clinical evidence
Jiang Y et al.	2024	N/A	Systematic review	Diagnosis/Stratification: synthesises TS biomarkers evidence base—supports biomarker-driven features for future AI models.	Heterogeneity; depends on included studies	TS-specific evidence synthesis
de Barros et al.	2025	TS (*N* = 34); Normal (*N* = 34)	Retrospective observational	Diagnosis: MRI texture features + ML association signals potential imaging-derived features for TS models.	Small dataset; age-range effects not analysed; sex differences not assessed	TS-specific, early clinical evidence
Zhang Z et al.	2025	*N* = 157	Retrospective observational	Prognosis/Stratification (cross-domain exemplar): multimodal radiomic–immunologic scoring illustrates fusion strategies transferable to TS precision stratification logic.	Not TS	Cross-domain exemplar
Higgins K et al.	2025	*N* = 38,049	Retrospective observational	Treatment (cross-domain exemplar): RL “AI-clinician” personalises regimen recommendations; supports “algorithm-guided personalisation” narrative for TS.	Not TS; prospective impact in TS unknown	Cross-domain exemplar (externally validated)
Saadullah Khani S et al.	2024	N/A	Systematic review	Treatment (indirect): summarises pharmacogenetic testing to guide antipsychotics—supports AI/PGx-informed individualised medication selection concept.	Mixed evidence; more RCTs needed	Indirect (psychiatry), not TS-specific
van de Mortel L et al.	2025	*N* = 159	Retrospective observational	Treatment/Prognosis (cross-domain exemplar): ML predicts CBT outcome using clinical+neuroimaging—supports “predictive models for treatment response” framing.	Not TS; details NR	Cross-domain mental health exemplar
Jöhnk M et al.	2025	30	RCT study (pilot)	Treatment/Delivery: digital app–assisted behavioural treatment improves scalability and structured follow-up; enables data capture for future “intelligent” personalisation.	Small pilot; larger RCT needed	TS/CTD, clinically tested (pilot RCT)
Andrén P et al.	2024	*N* = 221	RCT study (follow-up)	Treatment/Long-term management: internet-delivered ERP with follow-up supports remote, continuous care pathway (foundation for monitoring + adaptive support).	No between-group superiority; possible natural symptom improvement and co-interventions confounding	TS/CTD, clinically tested
Balachandar R et al.	2024	N/A	Review	Treatment/Monitoring: reviews closed-loop DBS—core concept for AI-enabled adaptive neuromodulation.	Review; TS-specific efficacy not established	Under development (TS-indirect)
Guo D; Choo KR	2025	N/A	Scoping review	Safety/Treatment optimisation (cross-domain): federated LLMs for ADR prediction—supports privacy-preserving learning for medication safety.	Not TS; scoping review	Cross-domain methods review
Dutta A; Das A	2025	N/A	Protocol	Treatment/Monitoring (cross-domain): XR biofeedback at-home platform concept—supports remote intervention infrastructure (no efficacy yet).	Protocol only; not TS	Under development (protocol)
Jo S et al.	2024	*N* = 45	RCT study	Treatment experience (cross-domain): immersive VR therapy improves function/satisfaction in rehab settings—supports engagement rationale for TS adjuncts.	Not TS; metrics NR	Cross-domain exemplar
Chen L et al.	2025	*N* = 101	Retrospective observational	Prognosis (cross-domain exemplar): DL multimodal fusion for recurrence prediction—supports “multimodal fusion improves prediction” logic.	Not TS	Cross-domain exemplar
Green M et al.	2025	*N* = 2,135	Retrospective observational	Monitoring/Treatment (cross-domain): AI-software–dependent neuromodulation with remote monitoring in real-world setting supports feasibility of monitored, adaptive therapy models.	Not TS	Cross-domain exemplar
Uthman OA et al.	2025	N/A	Overview	Economic/implementation (cross-domain): evidence-synthesis synopsis illustrates how prevention strategies are evaluated for effectiveness/cost—template for TS cost-effectiveness framing.	Not TS; overview scope	Cross-domain evidence synthesis

#### 5.4.1 Current status of AI in TS

Specific progress in TS is most evident in two areas: automated diagnostic assessment and digital therapeutic delivery. Studies utilising computer vision and deep learning have demonstrated the feasibility of objective tic quantification, potentially resolving the bottleneck of subjective human rating. Similarly, pilot randomised controlled trials (RCTs) of app-assisted behavioural interventions confirm that digital platforms can effectively deliver evidence-based treatments like CBT and ERP. However, robust AI-driven systems for prognostic prediction and pharmacogenomic personalisation in TS are still largely in the retrospective observational phase, relying on single-centre datasets without widespread external validation.

#### 5.4.2 Relevance of cross-domain evidence

To demonstrate the potential of precision medicine for TS, we cited advanced examples from fields like oncology and cardiology where AI is more established. These “exemplar” studies demonstrate how multimodal fusion and reinforcement learning can optimise outcomes in complex, heterogeneous conditions. While not direct evidence for TS, they serve as methodological benchmarks, validating the technical pathways that future TS research should emulate.

#### 5.4.3 Methodological critical analysis

A critical assessment of the cited methodology highlights several limitations that must be addressed to accelerate translation. The majority of TS-specific AI studies are retrospective observational studies with relatively small sample sizes and often lack independent testing sets, unlike the large-scale, externally validated cohorts seen in oncology, raising concerns about overfitting. Furthermore, many diagnostic models rely on “ground truth” labels derived from subjective clinical assessments or controlled video segments that may not capture naturalistic symptom fluctuations. Finally, despite innovative frameworks proposed in methodological reviews, there is a paucity of prospective RCTs specifically designed to evaluate the clinical utility of AI decision-support tools compared to standard care in TS.

In conclusion, while the technological feasibility of AI in TS is well-supported, establishing its clinical value will require a shift from small, retrospective pilots to large-scale, multi-centre prospective validation, guided by the rigorous standards observed in more mature precision medicine fields.

## 6 Technical challenges, solutions, and future outlook

Notwithstanding the considerable potential and application prospects of AI-driven precision medicine for TS, numerous technical challenges persist in terms of practical implementation. The subsequent chapter will methodically analyse these challenges, propose corresponding solutions, and consider future development directions.

### 6.1 Technical challenges

#### 6.1.1 Data quality challenges

High-quality data is fundamental for AI in TS precision medicine, yet significant challenges persist. Firstly, there is a paucity of complete and consistent TS-related medical data, which is further compounded by the presence of extraneous information (Sharifnia et al., 2025). Secondly, data standards vary among different medical institutions, and data formats differ significantly, making effective data integration difficult (Ho et al., 2026). Thirdly, TS symptoms are volatile and heterogeneous, making standardised data collection difficult (Ortiz et al., 2024). Data quality challenges also manifest in the accuracy of data annotation. The evaluation of symptoms in TS is predominantly determined by the subjective assessment of clinicians, which can result in variations among different physicians, thereby impacting the precision of data annotation (Gut et al., 2024). Furthermore, the symptoms of TS are complex and diverse, which makes it difficult to accurately annotate all symptom types and severity.

#### 6.1.2 Algorithm interpretability challenges

Algorithm interpretability is a prerequisite for clinical adoption but remains challenging. Firstly, deep learning algorithms are often ‘black boxes,’ making their decision-making processes opaque (Gujral et al., 2025). Secondly, the pathological mechanisms of TS are complex, involving multiple neurotransmitter systems and brain regions, requiring AI algorithms’ decision basis to be combined with neuroscience theories (Alkam et al., 2025). Thirdly, clinicians must develop a comprehensive understanding of the decision-making processes employed by AI algorithms to ensure accurate and effective clinical judgements in practice (Agard et al., 2025). Algorithm interpretability challenges also manifest in the accuracy of model explanation. Although explanation methods such as SHAP can provide feature importance analysis, further verification is required regarding their accuracy and reliability (Esan et al., 2025). Furthermore, the selection of an explanation method may yield divergent results, thus emphasising the necessity of identifying the most appropriate method.

#### 6.1.3 Clinical translation challenges

The translation of AI technology from a laboratory context to clinical application is encumbered by numerous challenges. Firstly, it should be noted that the performance of AI models in laboratory settings may differ from that in real clinical environments (Agard et al., 2025). Secondly, the complexity and uncertainty of the clinical environment may affect the performance of AI models (Lin et al., 2025). Thirdly, the clinical application of AI technology necessitates integration with existing medical processes and systems, thereby increasing the complexity of translation (Steinhauser and Welsch, 2025). Regulatory and certification requirements also complicate clinical translation. The utilisation of AI in medical devices necessitates the attainment of pertinent regulatory certifications, a process that entails meticulous clinical trials and rigorous safety verification (Hussain et al., 2025). Moreover, the clinical implementation of AI technology necessitates the establishment of quality control and quality assurance systems to ensure the safety and efficacy of the technology (Bönisch et al., 2025).

#### 6.1.4 Ethical and legal challenges

AI implementation in TS precision medicine raises critical ethical and legal concerns. Firstly, patient privacy protection is a crucial concern, as AI technology needs to process large amounts of personal health data, and how to protect patient privacy is a challenge (Konnerth et al., 2025). Secondly, concerns regarding fairness and bias in AI algorithms necessitate consideration to ensure that AI technology does not exhibit discriminatory tendencies (Bull et al., 2025). Thirdly, the issue of AI technology accountability requires elucidation, as the allocation of responsibility when AI systems err is a challenge (Giorgetti et al., 2025). The ethical and legal challenges experienced in this context manifest in issues pertaining to informed consent and autonomous decision-making. Patients should be provided with clear, accessible information about the role, limitations, and potential risks of AI-assisted tools to support informed consent (Allen et al., 2025). Furthermore, it is imperative to ensure that the implementation of AI technology does not compromise patients’ autonomy in making decisions regarding their care. A harmonious balance must be achieved between the utilisation of technological assistance and the preservation of patient autonomy (Ancillotti et al., 2025).

### 6.2 Solutions

#### 6.2.1 Multi-centre collaboration and federated learning

Multi-centre collaboration effectively addresses data quality issues. The establishment of a multi-centre collaborative network facilitates the integration of data from diverse medical institutions, thereby enhancing data diversity and representativeness (Shimizu et al., 2025). Furthermore, multi-centre collaboration has been demonstrated to promote the unification of data standards, thus improving data quality. Federated learning is an emerging machine learning method that enables the training of models while protecting data privacy (Dong et al., 2025). Federated learning facilitates collaboration among medical institutions for the purpose of training artificial intelligence (AI) models without the need for the sharing of raw data. This approach has been shown to address concerns regarding data privacy protection while concomitantly enhancing the generalisability of the models (Song et al., 2026). The combination of multi-centre collaboration and federated learning has the potential to provide substantial data support for TS precision medicine. The establishment of a TS multi-centre collaborative network facilitates the integration of data from diverse regions and medical institutions, thereby enhancing the performance and reliability of AI models (Kamelia et al., 2025).

#### 6.2.2 Explainable AI and SHAP analysis

Explainable AI (XAI) addresses interpretability challenges by clarifying AI decision-making. XAI technologies have the capacity to furnish clinicians with the rationale underlying the decision-making process of AI systems, thereby facilitating a more profound comprehension of the decision-making basis of AI algorithms (Agard et al., 2025). Commonplace XAI technologies include LIME, SHAP, Grad-CAM, among others (Akgündoğdu and Çelikbaş, 2025). SHAP (SHapley Additive exPlanations) represents a significant advancement in the field of XAI technology, with the capacity to quantify the contribution of each feature to model predictions (Anand et al., 2025). The application of SHAP analysis facilitates the identification of pivotal factors influencing TS prediction, thereby providing a scientific foundation for clinical decision-making processes. Recent TS-specific work has used SHAP to highlight influential predictors in risk prediction models, including β2-microglobulin and serum 25-hydroxyvitamin D (Li et al., 2025). The integration of explainable artificial intelligence (AI) with SHAP analysis has the potential to facilitate transparent decision support for precision medicine in the field of TS. SHAP analysis helps clinicians understand AI decisions, fostering trust (Ranwala and Andrade, 2025).

#### 6.2.3 Standardisation and regulatory frameworks

Standardisation is pivotal for clinical translation. The establishment of technical, data, and quality standards for TS precision medicine is imperative to ensure the normalisation and standardisation of technology (Vogel et al., 2025). Standardisation can also promote interoperability between different systems, improving the portability of technology. Regulatory frameworks are essential to ensure AI safety and efficacy. It is imperative to establish regulatory frameworks that are conducive to the integration of AI medical technology, encompassing technology evaluation, clinical trials, and market access (Hogg et al., 2025). The establishment of regulatory frameworks must strike a balance between the encouragement of technological innovation and the assurance of patient safety. The combination of standardisation and regulatory frameworks has the potential to provide institutional guarantees for the clinical application of TS precision medicine. The establishment of comprehensive standards and regulatory systems is imperative for ensuring the safety and effectiveness of AI technology, thereby facilitating its clinical translation (Barrett et al., 2025).

#### 6.2.4 Ethical guidelines and legal frameworks

Robust ethical guidelines are needed to address legal and ethical challenges. It is imperative to establish ethical guidelines that are compatible with AI medical technology, encompassing requirements for patient privacy protection, algorithm fairness, and informed consent (Ahmed et al., 2025). In addition, multidisciplinary consensus-driven reporting standards for generative AI systems (e.g., chatbot health advice studies) have been proposed to improve transparency and accountability, typically involving broad stakeholder engagement during guideline development (Chart Collaborative, 2025). The enhancement of legal frameworks constitutes a pivotal assurance for ensuring the compliant application of AI technology. Legislation must be enhanced to clarify AI accountability and data protection (Alanazi, 2025). The enhancement of legal frameworks must demonstrate adaptability to technological developments, with the dual objectives of safeguarding patient rights and fostering technological innovation. The combination of ethical guidelines and legal frameworks has the potential to provide ethical and legal guarantees for the clinical application of TS precision medicine. The establishment of comprehensive ethical guidelines and legal frameworks is imperative to ensure the compliant application of AI technology and to protect patient rights (Liang, 2025).

### 6.3 Future development directions

#### 6.3.1 Technical development directions

The technical development directions of AI technology in TS precision medicine primarily encompass the optimisation of algorithms, the integration of multimodal data, and the implementation of real-time processing. Firstly, it is hypothesised that the development of algorithms will be characterised by an enhancement in efficiency, accuracy, and interpretability (Anand et al., 2025). Secondly, multimodal fusion technology will become more mature, capable of integrating a greater variety of data (Tran et al., 2025). Thirdly, advancements in real-time processing technology will facilitate the attainment of millisecond-level data processing and decision response capabilities (Kolivand et al., 2025). The development of emerging technologies will provide new technical support for TS precision medicine. For instance, quantum computing technology has the potential to offer novel computational capabilities for the analysis of complex data (Idress et al., 2025). Edge computing technology has the potential to offer novel technical solutions for real-time monitoring and automatic adjustment (Saratkar et al., 2025). The potential of blockchain technology to provide novel solutions for data security and privacy protection is a subject of considerable interest (Selvi and Sakthivel, 2025).

#### 6.3.2 Clinical application directions

The clinical applications of AI technology in TS precision medicine are expected to evolve towards a more comprehensive, nuanced, and personalised approach. Firstly, the scope of application will be broader, extending from diagnosis to treatment, monitoring, and prognostic assessment (Yi and Cho, 2025). Secondly, the depth of application will be deeper, extending from single functions to comprehensive intelligent diagnostic and therapeutic systems (Zubair et al., 2025). Thirdly, the degree of personalisation will be higher, capable of providing customised diagnostic and therapeutic services for each patient (Liu X. et al., 2025). The development of clinical applications is set to drive the transformation of TS diagnostic and therapeutic models. Conventional “one-size-fits-all” diagnostic and therapeutic models are anticipated to undergo a transition towards individualised precision diagnostic and therapeutic models (Breunis et al., 2025). Conventional empirical diagnosis and treatment will undergo a transition towards data-driven intelligent diagnosis and treatment (Moralez et al., 2025). Conventional passive diagnosis and treatment will undergo a transition towards active prevention and intervention (Tasmurzayev et al., 2025).

#### 6.3.3 Industrial development directions

The AI-driven precision medicine industry is expected to evolve in a manner that is increasingly characterised by maturity, standardisation, and sustainability. Firstly, the industrial ecosystem will be more complete, including various participants such as technology providers, medical institutions, regulatory bodies, and patient organisations (Abozaid et al., 2025). Secondly, business models will be more mature, incorporating a variety of models such as technology services, data services and platform services (Kurz et al., 2025). Thirdly, the industrial chain will be more complete, providing full-chain services from technology research and development to clinical application (Gomase et al., 2025). The implementation of TS precision medicine on a large scale is expected to be significantly influenced by industrial development. The advent of industrialisation has been instrumental in reducing the financial burden associated with technological advancements, thereby enhancing their accessibility, a development that has been shown to have a positive impact on a greater number of TS patients (Gruia et al., 2025). Furthermore, the process of industrial development has been demonstrated to have the capacity to promote technological innovation and the continuous progression and enhancement of technology (Montalban et al., 2025).

### 6.4 System architecture and implementation path

#### 6.4.1 Data acquisition and processing architecture

Robust data architecture is the foundation of AI-driven TS systems. The establishment of a multi-level data acquisition system is imperative, encompassing clinical data, imaging data, biomarker data, behavioural data, and related domains (Peng et al., 2021). In order to ensure data quality and consistency, it is essential that data acquisition is standardised and normalised (Baumgartner et al., 2024). The data processing architecture must be capable of supporting the fusion and processing of multimodal data. The establishment of data preprocessing, feature extraction, and data fusion processes is imperative (Mahmood et al., 2025). The data processing architecture must also be capable of supporting both real-time processing and batch processing in order to meet the requirements of the various application scenarios (Arshad et al., 2025). Data security architecture constitutes a pivotal component of the data acquisition and processing architecture. It is imperative that a comprehensive data security protection mechanism be implemented, encompassing data encryption, access control, and audit logs (Wassan et al., 2025). It is imperative that the data security architecture complies with the relevant legal and regulatory requirements in order to ensure the protection of patient privacy (Kaschta et al., 2025).

#### 6.4.2 Algorithm engine design

Algorithm engine design constitutes the fundamental aspect of AI-driven TS precision medicine systems. The establishment of a modular algorithm engine is imperative to support the diverse range of machine learning algorithms and deep learning models (Zafar and Zafar, 2025). The algorithm engine must support the full lifecycle management of models, including training, validation, deployment, and updating (Bilal et al., 2025). The algorithm engine must be capable of supporting the processing and analysis of multimodal data. The establishment of specialised multimodal fusion algorithms is imperative for the integration of data from disparate sources (Yang et al., 2025). Furthermore, the algorithm engine must be capable of supporting both real-time inference and batch inference in order to satisfy the diverse requirements of various application scenarios. The interpretability of the algorithm engine is a significant design consideration. The integration of explainable AI technologies is imperative to facilitate the interpretation of algorithmic decisions (Yu et al., 2025). The algorithm engine must also facilitate the visualisation and analysis of models, thereby assisting users in comprehending the operational principles of the model (Ogundokun et al., 2025).

#### 6.4.3 Application service deployment

Application service deployment represents a pivotal component of AI-driven TS precision medicine systems. The establishment of a cloud-native application service architecture is imperative to facilitate elastic scaling and ensure high availability (Ileana et al., 2025). Supporting microservice architecture facilitates maintenance and upgrades (Islam et al., 2025). Services must support multiple deployment modes, including cloud, edge, and hybrid options (Islam et al., 2025). The deployment of these systems is contingent upon the selection of an appropriate mode, which is determined by the specific requirements of the given application scenario. The security of application services is a key consideration. Comprehensive security mechanisms, such as identity authentication and data encryption, are essential (Ileana et al., 2025). Security auditing and monitoring are also required to ensure safe operation (Palmer and Schwan, 2025).

#### 6.4.4 User interface design

The user interface design constitutes a pivotal element in facilitating seamless interaction between AI-driven TS precision medicine systems and their users. The design of an intuitive, easy-to-use, and aesthetically pleasing user interface is imperative to enhance the user experience (Del Piccolo et al., 2025). The user interface must be capable of supporting multiple devices, encompassing desktops, mobile devices, and tablets (Bernardelli et al., 2025). The user interface must cater to the diverse requirements of different user roles, including doctors, patients, and administrators (Abbasi et al., 2024). Designers must account for diverse user roles, creating tailored interfaces for doctors, patients, and administrators. Consequently, the design of corresponding interfaces and interaction methods is paramount. The accessibility of the user interface is a significant design consideration. Barrier-free access is necessary to ensure inclusivity for all users (Desmond et al., 2018). The user interface must also support multiple languages in order to meet the needs of users in different regions (Hussein et al., 2004).

#### 6.4.5 System integration and optimisation

The successful implementation of AI-driven TS precision medicine systems is contingent upon system integration and optimisation. A comprehensive integration architecture is required for seamless connection with existing medical systems (Bastola et al., 2025). Integration must adhere to relevant standards to ensure interoperability (Guru Rao et al., 2025). System optimisation should be a continuous process, covering performance, functionality, and user experience (Howard-Williams et al., 2025). Optimisation of the system must be based on user feedback and data analysis in order to ensure continuous improvement in system performance and functionality (Jiao et al., 2025). System monitoring and maintenance are integral components of optimisation. A comprehensive monitoring system ensures real-time surveillance of operational status (Ayouni et al., 2025). Maintenance procedures must be standardised to ensure the stability of the system’s operation (Hua et al., 2025). In summary, AI-driven TS precision medicine faces technical challenges, but these challenges can be overcome through efforts in technological innovation, institutional construction, and talent cultivation. In the future, the development of AI-driven TS precision medicine will proceed in the direction of increased intelligence, personalisation and precision, thus providing higher quality medical services for patients with TS. The establishment of a comprehensive system architecture and implementation strategy is imperative for ensuring the effective application of AI technology and the sustainable development of TS precision medicine.

## 7 Conclusion

This perspective outlines the transformative potential of AI-driven precision medicine for Tourette syndrome (TS). We synthesise current research trends to highlight clinical opportunities, limitations, and priorities for translation. Overall, AI offers meaningful technical advantages for TS precision medicine, particularly in data integration, prediction, and continuous monitoring. Specifically, multimodal diagnostic models may substantially improve diagnostic performance by integrating complementary clinical, behavioural, and neurobiological signals, as suggested by progress in other complex medical domains. Furthermore, pharmacogenomics-informed medication selection may improve treatment stratification and reduce avoidable adverse reactions, although TS-specific effectiveness must be validated in robust clinical studies. Finally, intelligent treatment systems incorporating real-time monitoring and adaptive adjustment may improve symptom control and support more responsive, patient-centred care. Collectively, these advances provide a foundation for more precise diagnosis and individualised management of TS, with the potential to improve outcomes as evidence and implementation mature.

The clinical impact of AI-driven TS precision medicine could be substantial. Diagnostically, multimodal fusion may reduce misdiagnosis by integrating diverse sources of evidence and enabling more objective assessment. Therapeutically, personalised strategies can tailor treatment plans to patient characteristics, comorbidities, and preferences, potentially improving tolerability and adherence. Prognostically, prediction models that integrate multi-source longitudinal data may support earlier risk stratification and targeted interventions.

Despite its promise, major technical and translational barriers remain for AI-driven TS precision medicine. Data quality challenges encompass a range of issues, including incomplete, inconsistent, and noisy data, as well as inconsistent data standards across various medical institutions. The challenges posed by interpretability in complex algorithms, such as deep learning, are characterised by their “black box” nature, which hinders clinicians’ ability to comprehend the decision logic of AI systems. The challenges associated with clinical translation include the performance discrepancies of AI models in real clinical environments and the complexity of integration with existing medical systems. The ethical and legal challenges associated with this practice encompass a range of issues, including the protection of patient privacy, the fairness of algorithms, and the establishment of accountability mechanisms. Future efforts must prioritise multi-centre collaboration, explainable AI (XAI) development, standardisation, and interdisciplinary cooperation.

Integrating AI into TS precision medicine represents a promising step towards more individualised, data-driven care. Achieving this vision will require high-quality multicentre datasets, clinically meaningful biomarkers, interpretable models, and rigorous prospective validation, alongside clear governance for privacy, fairness, and accountability. Prioritising interoperable data standards and practical clinical workflows will be essential to translate these tools from proof-of-concept to routine care.
